# Prediction of esophagogastric varices associated with oxaliplatin administration

**DOI:** 10.1002/jgh3.12668

**Published:** 2021-11-02

**Authors:** Yosuke Satta, Ryuta Shigefuku, Tsunamasa Watanabe, Takuro Mizukami, Takashi Tsuda, Tatsuya Suzuki, Takuya Ehira, Nobuhiro Hattori, Hirofumi Kiyokawa, Kazunari Nakahara, Hiroki Ikeda, Kotaro Matsunaga, Hideaki Takahashi, Nobuyuki Matsumoto, Chiaki Okuse, Michihiro Suzuki, Yu Sunakawa, Hiroshi Yasuda, Fumio Itoh

**Affiliations:** ^1^ Division of Gastroenterology and Hepatology St. Marianna University School of Medicine Kawasaki Kanagawa Japan; ^2^ Department of Gastroenterology and Hepatology Mie University Graduate School of Medicine Tsu Japan; ^3^ Department of Clinical Oncology St. Marianna University School of Medicine Kawasaki Kanagawa Japan; ^4^ Center for Hepato‐Biliary‐Pancreatic and Digestive Disease Shonan Fujisawa Tokushukai Hospital Kanagawa Japan; ^5^ Division of Gastroenterology and Hepatology Kawasaki Tama Municipal Hospital Kawasaki Japan; ^6^ Division of Gastroenterology Yokohama City Seibu Hospital Yokohama Japan; ^7^ Division of General Internal Medicine, Department of Internal Medicine Kawasaki Tama Municipal Hospital Kawasaki Japan

**Keywords:** esophagogastric varices, oxaliplatin, porto‐sinusoidal vascular disease, splenomegaly, thrombocytopenia

## Abstract

**Background:**

Oxaliplatin is a key drug for the chemotherapy of colorectal cancer; however, it is also known to cause non‐cirrhotic portal hypertension. We aimed to identify the characteristics of patients who developed esophagogastric varices (EGVs) after treatment with oxaliplatin.

**Methods:**

This study retrospectively analyzed patients with colorectal cancer who were treated with chemotherapy including oxaliplatin between 2010 and 2016. All patients were evaluated by contrast‐enhanced computed tomography (CE‐CT) every 3 months both during and after treatment; and endoscopy was performed when appearance of portal hypertension was suspected.

**Results:**

A total of 106 patients were divided into two groups: EGV formation (*n* = 6) and EGV non‐formation (*n* = 100). In the EGV group, platelet counts decreased and the size of the spleen calculated by CT (CT spleen index; CT‐SI) increased markedly. The highest area under the receiver operating characteristic curve (AUC) for the change in platelet counts was 0.81 (80% sensitivity and 83% specificity) at 3 months post treatment, and the maximum AUC for CT‐SI was 0.89 (79% sensitivity and 83% specificity) at 6 months post treatment.

**Conclusions:**

EGV formation could be predicted by the assessment of platelet counts and spleen size. If progressive splenomegaly and thrombocytopenia are observed not only during but also after completion of the oxaliplatin‐containing chemotherapy, EGVs should be confirmed by endoscopy for avoiding subsequent rupture.

## Graphical abstract

We aimed to identify the characteristics of patients who developed esophagogastric varices (EGV) after treatment with oxaliplatin. If progressive splenomegaly and thrombocytopenia are observed not only during but also after completion of the oxaliplatin‐containing chemotherapy, EGV should be confirmed by endoscopy for avoiding subsequent rupture.

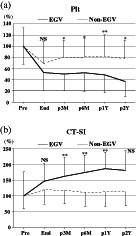

## Background

Oxaliplatin is one of the most commonly used chemotherapeutic drug in gastrointestinal cancers.^1^, ^2^ The addition of oxaliplatin or irinotecan to 5‐fluorouracil (5‐FU)/leucovorin therapy (FU/LV) has been shown to improve response rates, progression‐free survival, and overall survival in patients with stage III and IV or recurrent colorectal cancer.^3^ However, hepatotoxicity induced by oxaliplatin has been reported by surgeons^4^, ^5^, ^6^ and should be carefully considered along with liver function because of the high incidence of postoperative complications. Therefore, oxaliplatin is now recognized as a hepatotoxic drug, based on pathological evidence of sinusoidal endothelial injury in the liver.^7^ Recently, it has also become widely known that oxaliplatin may lead to the development of portal hypertension and esophagogastric varices (EGVs). Some case reports, including from our group, have shown that EGV formation or rupture during or after oxaliplatin‐containing chemotherapy can make it difficult to continue the treatment and may worsen the prognosis.^8^, ^9^, ^10^, ^11^, ^12^ However, the prevalence of and risk for developing EGVs after oxaliplatin treatment are unclear. We therefore evaluated clinical features predictive of the development of EGVs in colorectal cancer patients treated with oxaliplatin‐based chemotherapy.

## Methods

### Patients and regimen of oxaliplatin‐based chemotherapy

We retrospectively reviewed the clinical data for 203 consecutive patients with colorectal cancer, who were treated with systemic chemotherapy including oxaliplatin as first‐line therapy due to advanced or recurrent colorectal cancer between October 2010 and January 2016. Subjects included in the present study were chemotherapy‐naive patients who completed at least four cycles of initial chemotherapy including oxaliplatin, as well as patients who had never received previous chemotherapy.

The patients in this study were treated with 5‐FU, LV, and oxaliplatin (FOLFOX); FOLFOX plus bevacizumab (FOLFOX/Bev); FOLFOX plus cetuximab (FOLFOX/Cet); or FOLFOX plus panitumumab (FOLFOX/Pan). The FOLFOX regimen consisted of 85 mg/m^2^ oxaliplatin and 200 mg/m^2^ LV via 2‐h infusion, followed by a bolus injection of 400 mg/m^2^ FU and 46‐h infusion of 2400 mg/m^2^ FU. The FOLFOX/Bev regimen consisted of FOLFOX plus bevacizumab (5 mg/kg via 90‐min infusion on day 1). The FOLFOX/Cet regimen consisted of FOLFOX plus cetuximab (400 mg/m^2^ via 120‐min infusion on day 1 for the first cycle and 250 mg/m^2^ via 60‐min infusion thereafter). The FOLFOX/Pan regimen consisted of FOLFOX plus panitumumab (6 mg/kg via 60‐min infusion on day 1). The first‐line chemotherapy including oxaliplatin was continued in 2‐week cycles until disease progression or undue toxicity. Second‐line or third‐line therapy without oxaliplatin after failure of the first‐line oxaliplatin‐containing chemotherapy was continued until disease progression, undue toxicity, or death.

The protocol for the present study was approved by the local ethics committee of St. Marianna University School of Medicine, in accordance with the ethical standards specified in the 1964 Declaration of Helsinki and its later amendments, and after written informed consent was obtained from all patients (Approval No. 4897).

### Data collection and assessments

As routine care, all patients underwent blood sampling for analysis of aspartate aminotransferase (AST), alanine transaminase (ALT), bilirubin, albumin, and platelet count every 2 weeks, and were evaluated by contrast‐enhanced computed tomography (CE‐CT) every 3 months both during and after treatment with oxaliplatin. If portal hypertension (i.e., collateral vessels, spleen enlargement, and EGVs on CE‐CT) was suspected, esophagogastroduodenoscopy (EGD) was performed.

### Spleen size (CT spleen index)

Spleen size was measured by helical CT scan at areas of the axial portal venous phase images created by consecutive sequential 5‐mm‐thick slices. The CT spleen index (CT‐SI) was calculated based on the maximum width and thickness at the hilum of the spleen on CT.^13^ In brief, measurements of the maximum width of the spleen and the thickness at the hilum determined on a plane perpendicular to the maximum splenic width and through the hilum were multiplied. Changes in spleen size were determined for each CT time point during and after therapy by comparison with the pre‐treatment value.

### Statistical analyses

Data are presented as the median with range for continuous data and numerically for categorical data. Continuous variables were compared between groups using non‐paired *t*‐test or Mann–Whitney *U*‐test. Categorical variables were compared between groups using Fisher's exact probability test. Cut‐off values were calculated using the Youden index for receiver operating characteristic (ROC) analysis. All *P*‐values for statistical tests were two‐tailed, and values of *P* < 0.05 were considered statistically significant. All statistical analyses were performed using Prism 5 software for Windows (GraphPad Software, Inc., La Jolla, CA, USA).

## Results

### Study cohort and patient characteristics

**Figure 1:**
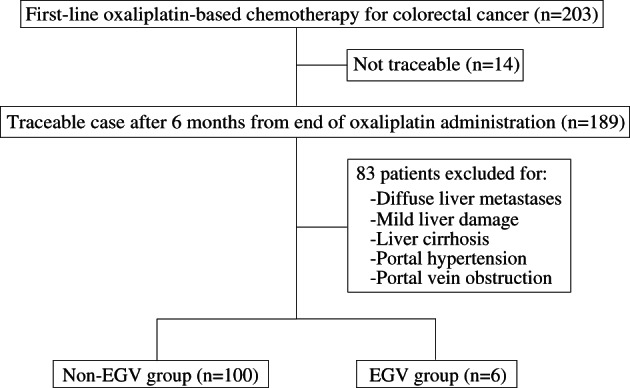
Patient flowchart. EGV, esophagogastric varice.

Of the 203 patients screened, 14 were excluded because CE‐CT scans had not been performed routinely, while 189 cases underwent complete follow‐up up to 6 months after the end of oxaliplatin‐based chemotherapy. Among these cases, 83 patients were excluded because of the presence of diffuse liver metastases, signs of mild liver damage (T‐Bil > 2.0 mg/dL or ALT > 100 IU/L) including liver cirrhosis, signs of portal hypertension (EGV and/or ascites), or portal vein occlusion before initial chemotherapy. Ultimately, 106 patients were included, and no patients had radiological signs of portal hypertension prior to chemotherapy. The cohort was divided into two groups with or without EGVs based on CE‐CT and/or EGD: an EGV group (*n* = 6) and a non‐EGV group (*n* = 100). And clinical features of these groups were analyzed and compared retrospectively (Fig. 1).

### Comparison of baseline characteristics between patients with and without EGV formation

The clinical characteristics of the 106 patients treated with systemic chemotherapy containing oxaliplatin as first‐line therapy for colorectal cancer are summarized in Table 1. The group with EGV (*n* = 6) had a median age of 60 years (range 49–73) and comprised five males and one female. The group without EGV (*n* = 100) had a median age of 66 years (range 27–87) and comprised 69 males and 31 females. There was no significant difference in tumor location between the two groups. In the EGV group, 33% (*n* = 2) received FOLFOX and 67% (*n* = 4) received FOLFOX with Bev, an inhibitor of the vascular endothelial growth factor. Although bevacizumab has been suggested to protect against hepatic injury in patients treated with oxaliplatin‐based chemotherapy,^14^ the effect could not be confirmed in this study group. In the non‐EGV group, for the first‐line treatment, 61% (*n* = 61) received FOLFOX, 33% (*n* = 33) received FOLFOX with Bev, and 6% (*n* = 6) received FOLFOX with Cet or Pan, inhibitors of the epidermal growth factor. The concomitant use of Cet or Pan might not have side effects.^15^ The EGV and non‐EGV groups showed no apparent difference in the mean number of cycles (11 vs 12 cycles, respectively). As a result of disease progression, other chemotherapy following FOLFOX was continued in 51 patients (51%) without EGV and 5 patients (83%) with EGV. No significant differences were found between the two groups.

**Table 1:** Characteristics of patients treated with systemic chemotherapy containing oxaliplatin as first‐line treatment for colorectal cancer

	Non‐EGV (*n* = 100)	EGV (*n* = 6)	*P*‐Value^†^
Age (years, median)	66 (27–87)	60 (49–73)	0.184
Sex (male/female), *n* (%)	69/31 (69/31)	5/1 (83/17)	0.556
Location of tumor			
Rectum/colon/cecum (%)	43/55/2	33/ 67/ 0	0.665^‡^
Stage of disease (stage ΙΙΙ/stage IV/recurrence)	31/63/6	0/ 5/ 1	0.553^§^
Presence of metastasis			
Liver/ not liver	53/47	3/ 3	0.901
Therapeutic regimen of the first‐line chemotherapy			
FOLFOX	61 (61%)	2 (33%)	0.243^¶^
FOLFOX + Bev	33 (33%)	4 (67%)	
FOLFOX + Cet or Pan	6 (6%)	0 (0%)	
Administration of oxaliplatin‐based chemotherapy			
Number of therapeutic cycles (times, median)	11 (3–44)	12 (9–29)	0.110
Other chemotherapy followed by FOLFOX			
Additional therapy (second‐ and third‐line chemotherapy)	51 (51%)	5 (83%)	0.163

^†^ Mann–Whitney *U*‐test.

^‡^ Rectum vs colon and cecum.

^§^ FOLFOX vs FOLFOX+Bev, Cet, or Pan.

^¶^ Stage III and stage IV vs recurrence.

Bev, bevacizumab; Cet, cetuximab; EGV, esophagogastric varices; FOLFOX, 5‐fluorouracil, leucovorin, and oxaliplatin; Pan, panitumumab.

### Liver damage caused by oxaliplatin‐based chemotherapy

**Figure 2:**
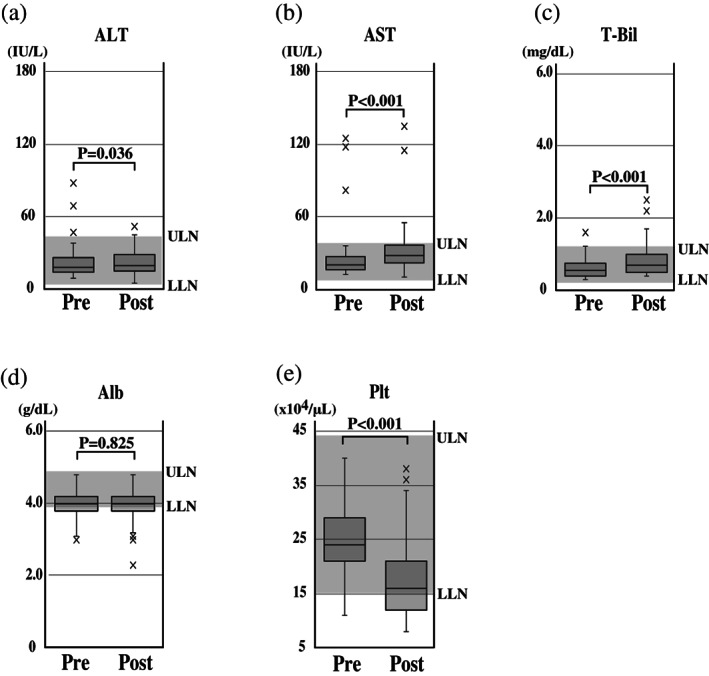
Laboratory values before and after oxaliplatin‐containing chemotherapy. The mean serum ALT, AST, and total bilirubin (T‐Bil) values were significantly increased post treatment but were within normal ranges (a–c). The mean albumin (Alb) level before and after chemotherapy did not differ significantly (d). However, the mean platelet count (Plt) was significantly decreased after chemotherapy and displayed apparent clinical significance (e). The gray areas represent the normal range. ULN, upper limits of normal; LLN, lower limits of normal.

None of the 106 patients included in the evaluation had laboratory signs of liver damage before chemotherapy: that is, elevated serum ALT, AST, total bilirubin, or albumin levels, or abnormal platelet counts. Following completion of oxaliplatin‐based chemotherapy, serum ALT, AST, and total bilirubin levels were significantly increased but were within the normal range (Fig. 2a–c). There was no significant difference in serum albumin levels before versus after chemotherapy (Fig. 2d). However, platelet counts were significantly decreased and the interquartile range of platelet counts at the end of oxaliplatin administration was not completely within the normal limits (Fig. 2e).

Collectively, the data showed no clinical evidence of liver damage induced by oxaliplatin‐containing chemotherapy, but the treatment did appear to have an effect on platelet counts.

### Features of cases that developed EGVs following oxaliplatin administration

As confirmed by EGD, six patients developed EGVs detected at a median of 25 months (range, 0–47 months) after the end of oxaliplatin administration; of these, four had received FOLFOX/Bev and two FOLFOX for the first‐line chemotherapy (Table 2). The six patients had received a median of 12 cycles (range 9–29 cycles) of oxaliplatin‐based chemotherapy at a median total oxaliplatin dosage of 1680 mg (range 1170–3770 mg).

**Table 2:** Dose and periods of oxaliplatin in esophagogastric varices (EGV) cases

No.	Age/sex	Chemotherapy regimen	Oxaliplatin total dose (mg/body)	Dose of oxaliplatin (mg/m^2^)	Therapeutic cycles of oxaliplatin (times)	Therapeutic periods of oxaliplatin (months)	Interval from the end of oxaliplatin to EGV detection (months)	EGV rupture (location)
1	49/F	First: FOLFOX + Bev Second: FOLFIRI + Bev Third: regorafenib	2600	2131	26	17	0	+ (gastric)
2	68/M	First: FOLFOX + Bev Second: FOLFIRI + Pan Third: CPT‐11 + Pan	1680	999	12	5	9	+ (esophageal)
3	60/M	First: FOLFOX + Bev Second: FOLFIRI + Ram Third: Regorafenib	1680	1002	12	7	19	+ (esophageal)
4	59/M	First: FOLFOX + Bev Second: 5‐FU/leucovorin + Bev Third: FOLFIRI + Bev	1240	793	9	6	30	− (esophageal)
5	73/M	First: FOLFOX	1170	841	10	5	46	− (esophageal)
6	57/M	First: FOLFOX second: FOLFIRI + Bev Third: HAI of 5‐FU Fourth: CPT‐11 + Cet Fifth: xeloda Sixth: Pan	3770	2432	29	16	47	− (gastric)
		Median	1680	1001	12	7	25	

There were two cases with ruptured esophageal varices (EV) who underwent endoscopic variceal ligation, two cases with unruptured EV who underwent endoscopic injection sclerotherapy, one case with unruptured gastric varices (GV) who underwent balloon‐occluded retrograde transvenous obliteration (B‐RTO), and one case with ruptured GV who underwent B‐RTO. There was no variceal rupture‐related death, four dead cases due to primary disease, and two cases with EGV still alive under treatment intervention.

Bev, bevacizumab; FOLFIRI, leucovorin, 5‐FU, and irinotecan (CPT‐11); FOLFOX, 5‐fluorouracil (5‐FU), leucovorin, and oxaliplatin; HAI, hepaticarterial infusion; Pan, panitumumab; Ram, ramucirumab.

Among the cases with EGV, the serum levels of ALT, AST, total bilirubin, and albumin before and after oxaliplatin‐containing chemotherapy showed almost no change, but platelet counts were decreased and CT‐SI was markedly increased (data not shown). At the point of EGV detection, either during or after treatment with oxaliplatin, the serum levels of ALT, AST, and total bilirubin were also within the normal range. Moreover, as evaluaed by CE‐CT, no morphological changes such as liver atrophy or swelling and ascites were observed at the time of EGD, except for EGV rupture cases with ascites.

### Change in platelet counts and CT‐SI

**Figure 3:**
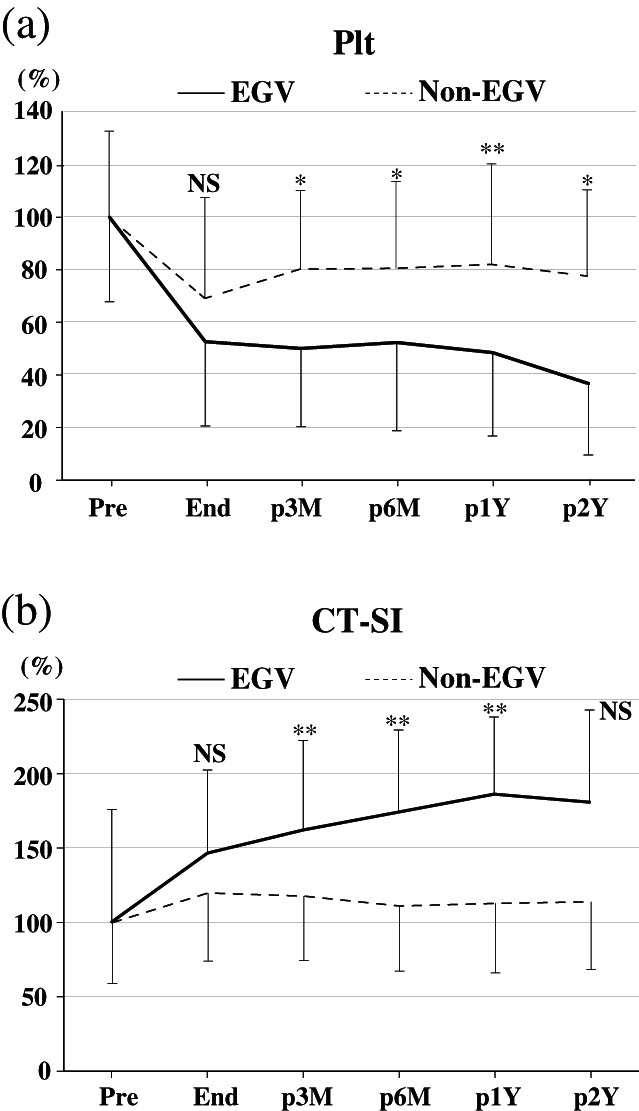
Platelet counts and CT spleen index in patients treated with oxaliplatin‐based chemotherapy. (a) Changes in platelet counts in the esophagogastric varice (EGV) and non‐EGV groups. Although the platelet count recovered after cessation of oxaliplatin treatment in the non‐EGV group, it continued to decrease after treatment in the EGV group. (b) Change in CT spleen index (CT‐SI) in the EGV and non‐EGV groups. Although CT‐SI improved after the end of oxaliplatin treatment in the non‐EGV group, it continued to increase after treatment in the EGV group. End, end of chemotherapy; NS, not significant; p1Y, after 1 year post treatment; p2Y, after 2 years post treatment; p3M, after 3 months post treatment; p6M, after 6 months post treatment; Pre, pre‐chemotherapy. *P < 0.01, **P < 0.001.

Platelet counts during oxaliplatin‐based chemotherapy are shown in Figure 3a. Although the platelet count in the non‐EGV group seemed to recover slightly after oxaliplatin treatment, the EGV group showed no recovery and the counts progressively decreased after completing oxaliplatin administration, with more than half the cases receiving other chemotherapy followed by oxaliplatin‐containing chemotherapy in both groups (Table 1). At every time point after 3 months post oxaliplatin treatment, change in platelet counts differed significantly among the two groups (*P* < 0.01). Similarly, the change in CT‐SI from during to after oxaliplatin treatment also significantly differed between the two groups (*P* < 0.001) except at 2 years post treatment (Fig. 3b).

The changes in platelet counts and CT‐SI in the six patients who developed EGVs compared with the 100 patients without EGVs indicated that the continuous decrease in platelet counts and/or increase in CT‐SI after oxaliplatin administration could signify the development of EGVs.

**Figure 4:**
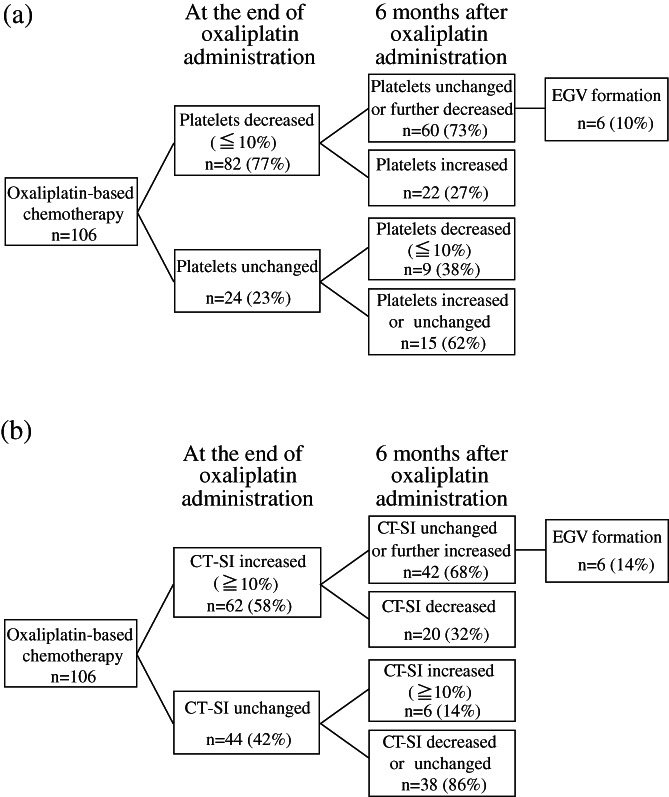
Occurrence of esophagogastric varice formation in patients receiving first‐line oxaliplatin‐based chemotherapy for colorectal cancer. (a) Change of platelet counts; (b) Change of CT spleen index. CT‐SI, CT spleen index; EGV, esophagogastric varices.

Flowcharts with details on EGV formation caused by oxaliplatin in relation to changes in platelet counts and CT‐SI are shown in Figure 4a,b. A decrease in platelet count of less than 10% and an increase in CT‐SI of more than 10% were discriminated at the end of and 6 months after the completion of oxaliplatin administration. As a result, EGV formation could develop only in cases of progressive splenomegaly and thrombocytopenia.

### Predictive factors for EGV formation

To predict EGV formation after oxaliplatin‐based chemotherapy, we calculated the cut‐off values, area under the curve (AUC), sensitivity, and specificity of the change in platelet counts and CT‐SI after oxaliplatin treatment using ROC analysis. The optimal cut‐off value, sensitivity, specificity, and AUC of changes in platelet counts for predicting EGV formation in response to oxaliplatin treatment are shown in Table 3. At the end of oxaliplatin‐based chemotherapy cut‐off of 62.5%, the sensitivity, specificity, and AUC were 61%, 67%, and 0.65, respectively. At the 3‐month post‐treatment cut‐off of 61.0%, the sensitivity, specificity, and AUC were 80%, 83%, and 0.81, respectively. At the 6‐month post‐treatment cut‐off of 64.0%, the sensitivity, specificity and AUC were 75%, 83%, and 0.79, respectively. Similarly, the optimal cut‐off value, sensitivity, specificity, and AUC of changes in CT‐SI for predicting EGV formation in response to oxaliplatin treatment are also shown in Table 3. At the end of oxaliplatin‐based chemotherapy cut‐off of 133%, the sensitivity, specificity, and AUC were 74%, 67%, and 0.77, respectively. At the 3‐month post‐treatment cut‐off of 128%, the sensitivity, specificity, and AUC were 73%, 83%, and 0.85, respectively. At the 6‐month post‐treatment cut‐off of 129%, the sensitivity, specificity, and AUC were 79%, 83%, and 0.89, respectively.

**Table 3:** Predicted values for EGV formation after oxaliplatin‐based chemotherapy using the change of platelet counts and computed tomography spleen index (CT‐SI)

Comparison with pre‐treatment value	Cut‐off value (% of pre‐treatment)	AUC	Sensitivity	Specificity
*Platelet counts after oxaliplatin‐based chemotherapy*				
End of chemotherapy	62.5	0.65	61	67
Post 3 months	61.0	0.81	80	83
Post 6 months	64.0	0.79	75	83
*CT‐SI after oxaliplatin‐based chemotherapy*				
End of chemotherapy	133	0.77	74	67
Post 3 months	128	0.85	73	83
Post 6 months	129	0.89	79	83

AUC, area under the receiver operating characteristic curve; EGV, esophagogastric varices.

Therefore, the response at 3 or 6 months post treatment, rather than immediately after the completion of oxaliplatin administration, could be a predictor for EGV formation caused by oxaliplatin‐based chemotherapy.

## Discussion

In this study, we found that EGV formation occurred after oxaliplatin administration in patients with progressive splenomegaly and thrombocytopenia. In contrast, EGV formation did not occur in patients with transient splenomegaly and/or thrombocytopenia (Fig. 3). Furthermore, we also found that platelet counts and CT‐SI were useful markers for predicting EGV formation caused by oxaliplatin‐based chemotherapy 3 or 6 months post treatment.

In general, drug‐induced liver injury (DILI) presents with extremely diverse histologic features, including necro‐inflammatory, cholestatic, steatotic, and vascular patterns. Vascular liver disease—also referred to as toxic sinusoidal injury, sinusoidal obstruction syndrome (SOS), or veno‐occlusive disease—comprises a commonly recognized vascular pattern of DILI.^7^ Consequently, it is well known that drug‐induced sinusoidal endothelial injury may be a consequence of the use of drugs such as busulfan, cyclophosphamide, azathioprine, and oxaliplatin, and non‐cirrhotic portal hypertension may develop subsequently.^16^ Recently, a group of experts on behalf of the Vascular Liver Disease Interest Group (VALDIG) proposed the term “porto‐sinusoidal vascular disease” (PSVD) to describe this condition.^17^ The definition of PSVD is based on the absence of cirrhosis with or without signs of portal hypertension or histologic lesions, and the term has been used to describe a form of idiopathic non‐cirrhotic portal hypertension. The epidemiology of this entity is unknown; however, the main etiologic factors identified in association with the development of PSVD are immunological disorders, infections, human immunodeficiency virus, drugs (azathioprine, oxaliplatin), toxins, genetic predisposition, and thrombophilia.^18^, ^19^, ^20^, ^21^ In agreement with a recent description,^17^ EGV formation after oxaliplatin treatment can be one of the clinical features of PSVD with portal hypertension.^22^

**Figure 5:**
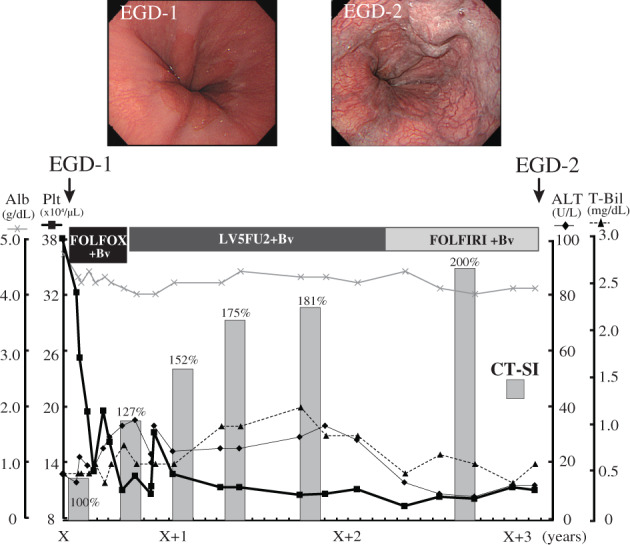
A case with esophagogastric varices formation after oxaliplatin administration. A 59‐year‐old Japanese man (case No. 4 in Table 2) received a diagnosis of esophagogastric varices (EGV) during treatment for sigmoid colon cancer. Evaluation by computed tomography (CT) revealed liver and lymph node metastases from a primary tumor in the sigmoid colon, the clinical stage of the tumor being stage IV. He had no relevant medical history and no EGV or splenomegaly before chemotherapy (shown in the upper left image). The patient underwent resection of the primary colonic tumor, and the pathological finding was stage IV colon adenocarcinoma (N1H2M1). He was then treated with nine cycles of FOLFOX (oxaliplatin, leucovorin, and 5‐fluorouracil) with bevacizumab as the first‐line chemotherapy for liver and lymph node metastasis. Because the treatment containing oxaliplatin (total oxaliplatin dosage was 1240 mg) was complicated by persistent thrombocytopenia and peripheral neuropathy at 6 months after completing the first‐line chemotherapy, LV5FU2 (leucovorin and 5‐fluorouracil) with bevacizumab, excluding oxaliplatin from FOLFOX, was continued. However, as his remaining cancer was diagnosed as progressive under FOLFOX and LV5FU2 therapy, chemotherapy was changed to a FOLFIRI (irinotecan, leucovorin, and 5‐fluorouracil) regimen. He was treated with systemic chemotherapy for over 3 years without hepatic dysfunction. To access the degree of EGV, esophagogastrodeuodenoscopy (EGD) was performed and EGV were detected (shown in the upper right image).

This study was retrospective and based on clinical manifestation of PSVD without a specific pathological confirmation. However, EGVs were accurately evaluated by EGD (i.e., a case shown in Fig. 5), and collateral vessel development, spleen enlargement, or EGV on CE‐CT was confirmed to have developed gradually and not to have been present before oxaliplatin administration. Our study, in agreement with a previous study,^22^ showed that PSVD leading to the development of portal hypertension is quite common in patients receiving oxaliplatin‐based chemotherapy. In our study cohort, 5.7% (6/106) of patients receiving oxaliplatin‐based chemotherapy developed apparent EGVs. However, the incidence rate of developing EGVs after oxaliplatin treatment was not determined because our study consisted of a small number of patients and had inclusion criteria aimed at excluding patients who were followed for less than 6 months from the end of oxaliplatin administration. Furthermore, while previous studies have reported that the development of non‐cirrhotic portal hypertension after oxaliplatin treatment seems to be dose‐dependent,^22^, ^23^ the data from our cohort showed that the development of EGV after treatment bears no obvious relationship with the total dosage of oxaliplatin. Further study is needed to address this point.

In our study, both platelet counts and CT‐SI tended to improve after the completion of oxaliplatin administration in the non‐EGV group. In contrast, progressive thrombocytopenia and splenomegaly occurred in the EGV group, indicating the possibility that the development of EGV can be predicted by evaluating platelet counts and CT‐SI. In the EGV group, there were no obvious changes in biochemical signs of liver damage, such as AST, ALT, and total bilirubin. Huang and colleagues suggested that oxaliplatin‐related portal hypertension is characterized by massive ascites, splenomegaly, gastric varices, concomitant arterio‐portal fistula, and relatively normal liver function.^11^ This report supported our data, indicating that the absence of signs of liver damage could delay the detection of EGVs. Therefore, it is important to assess changes in platelet counts and spleen size on CT scan in patients treated with oxaliplatin‐based chemotherapy.

The next focus of this study is the reversibility of oxaliplatin‐induced hepatotoxicity and/or EGV formation. Very littlle relevant data from such clinical studies have been published. Vigano et al. investigated the reversibility of liver injury after the interruption of chemotherapy and reported that SOS regressed only 9 months after the end of chemotherapy.^24^ Moreover, the M.D. Anderson Cancer Center's group surveyed the evolution of splenomegaly, which is associated with SOS, in 136 patients receiving prolonged oxaliplatin‐based chemotherapy; the condition with splenomegaly had regressed in 35% of the patients 6 months after the end of chemotherapy, in 85% at 1 year, and in all patients at 18 months.^23^ In contrast, a Japanese group reported that increased splenic volume had not recovered in 12 of 28 cases (42.9%) 1 year after completing oxaliplatin‐based chemotherapy.^25^ In our cohort, all EGV formation occurred in a group with progressive splenomegaly, but never regressed, after the end ofoxaliplatin administration. Therefore, the reversibility of oxaliplatin‐induced hepatotoxicity is still controversial, but EGV formation could occur in cases with progressive splenomegaly.

In this retrospective study, the limitations are the small sample size and the EGV screening procedure based on radiological findings prior to definitive diagnosis by endoscopy. Also, liver biopsy was not done to confirm the diagnosis of PSVD. We only evaluated patients with colorectal cancer who developed apparent EGVs due to oxaliplatin administration; so the incidence rate of developing EGVs may not be generalizable to patients treated with oxaliplatin‐based chemotherapy. Also, the existence of EGVs was not confirmed endoscopically in all cases because in a retrospective study based on reference to clinical data, usually no regular endoscopy is performed on all cases. Thus, prospective large‐scale trials with various diseases treated with oxaliplatin‐based chemotherapy, especially receiving adjuvant FOLFOX only, will be required in the future to clarify the effects of oxaliplatin administration on PSVD and EGV occurrence. Additionally, liver stiffness measurement, obtained using transient elastography or MR elastography, and hemodynamic studies might be required in future trails to assess and monitor the severity of oxaliplatin‐related portal hypertension.

Given that only six patients developed EGVs, the risk factors for developing EGV after oxaliplatin treatment could not be identified, but splenomegaly and thrombocytopenia, especially when occurring after the end of oxaliplatin administration, might be predictive of EGVs. Early detection of risk groups and evaluation by EGD in patients receiving oxaliplatin may prevent worsening of the prognosis associated with EGV rupture, and it might be possible to treat EGV using endoscopic therapy.

## Conclusions

Assessment of platelet counts and CT‐SI can enable the prediction of EGV formation not only during but also after completion of the oxaliplatin‐containing chemotherapy.

## Data Availability

The data sets used and/or analyzed during the current study are available from the corresponding author on reasonable request.
